# Standardization of techniques and conditions for *in vitro* ovulation induction in *Astyanax altiparanae*

**DOI:** 10.1590/1984-3143-AR2025-0003

**Published:** 2026-02-13

**Authors:** Cristiane Fernanda Benevente, Mariana Roza de Abreu, Queila Carla Ameno Alves, Laíza Maria de Jesus Silva, Roosevelt Passos Barbosa, Samyra Maria dos Santos Nassif Lacerda, Sergio Ricardo Batlouni

**Affiliations:** 1 Laboratório de Reprodução de Peixes, Centro de Aquicultura, Universidade Estadual Paulista – UNESP, Jaboticabal, SP, Brasil; 2 Instituto Capixaba de Pesquisa, Assistência Técnica e Extensão Rural – Incaper, Linhares, ES, Brasil.; 3 Departamento de Morfologia, Instituto de Ciências Biológicas, Universidade Federal de Minas Gerais – UFMG, Belo Horizonte, MG, Brasil

**Keywords:** fish ovulation, spawning induction, DHP, PGF_2alpha_, pituitary extracts

## Abstract

This study aimed to establish *in vitro* culture conditions for inducing ovulation in lambari (*Astyanax altiparanae*). All fish received a priming dose of either 0.6 mg/kg or 100 IU hCG/kg (human chorionic gonadotropin) *in vivo*. In experiment 1, 0.6 mg and 5.4 mg of carp pituitary extract (CPE)/ kg of ovarian fragment were tested as resolving doses. In experiment 2, prostaglandin F2α (PGF_2α_) analogue at 100 ng/mL was added to 5.4 mg CPE/kg as the resolving dose. Since ovulation did not occur, in experiment 3 and 4, we compared diverse forms of obtaining follicles, comparing manual follicle dissociation and collagenase at 100, 200, and 400 CDU/mL. Additionally, in experiment 4, the resolving dose of CPE and hCG was replaced by 1 µg/mL 17α,20β-dihydroxy-4-pregnen-3-one (DHP). Ovulation was successful only in Experiment 4, using mechanically dissociated follicles with a priming dose of either CPE or hCG and 1 µg/mL DHP as the resolving dose. Key findings include that 5% CO_2_ is unnecessary, mechanical dissociation of ovarian fragments is optimal, a priming dose of CPE or hCG is required, and DHP at 1 µg/mL is effective. These results establish a standardized protocol for *in vitro* ovulation induction in *A. altiparanae*, offering a valuable tool to study ovarian function and spawning failure in tropical species.

## Introduction

Spawning failures affect many species worldwide, leading to significant losses in aquaculture (Mylonas et al., 2010). One key advantage of *in vitro* studies is the ability to efficiently test a wide range of hormone types and doses in a short period. These systems enable researchers to gather data on a smaller, controlled scale (i.e., *in vitro*), reducing the need for live fish and minimizing hormone use. The direct application of hormonal agents *in vitro* cultures, which influence final oocyte maturation (FOM) and ovulation, has been widely used and has contributed to the development of effective protocols for various species produced globally (Jalabert and Szöllösi, 1975; Goetz and Theofan, 1979; Hagiwara et al., 2014; Tang et al., 2016, 2017). However, such approaches remain largely unexplored for native Latin American (LA) species. For lambari (*Astyanax altiparanae*), a small species important in aquaculture, especially as bait, and one for which the spawning rate requires enhancement (Roza de Abreu et al., 2021, 2022; Ariki et al., 2023), *in vitro* studies offer a valuable opportunity to test various hormones while reducing the need for euthanizing large numbers of individuals.

In recent years, we have demonstrated that a common issue in hypophysation processes is the successful resumption of meiosis, evidenced by germinal vesicle breakdown (GVBD) and oocyte maturation, but with no subsequent ovulation. GVBD oocytes often remain in the ovaries, a problematic phenomenon observed in species such as pacu (*Piaractus mesopotamicus*) (Criscuolo-Urbinati et al., 2012; Kuradomi and Batlouni, 2018; Sato et al., 2021 and 2023), matrinxã (*Brycon amazonicus*) (Hainfellner et al., 2012), piau três pintas (*Leporinus fridrici*) (Souza et al., 2020), piauçu (*Leporinus macrocephalus*) (Pereira et al., 2018), and lambari (*Astyanax altiparanae*) (Roza de Abreu et al., 2021, 2022; Ariki et al., 2023).

Ovulation, defined as the rupture of the ovarian follicle and release of a mature oocyte into the ovarian ducts (Jalabert, 2005), is mediated by luteinizing hormone (LH), which regulates the expression of nuclear progesterone receptors (*nPR*) and prostaglandin receptors (*ptger4b*) (Tang et al., 2016, 2017). Prostaglandins play a critical role in ovulation in fish (Hagiwara et al., 2020), and their synthesis occurs during both natural and induced ovulation in non-mammalian vertebrates (Takahashi et al., 2013). Much of the understanding of their hormonal function has been derived from studies about hormonal function comes from studies on species like carp (Jalabert et al., 1977; Levavi-Zermonsky and Yaron, 1986), trout (Bobe et al., 2006), and yellow perch (El Mohajer et al., 2021).

Based on this knowledge, we proposed modifying the conventional hypophysation protocol by incorporating prostaglandin F_2α_ (PGF_2α_), a potent inducer of follicle rupture (review in Batlouni et al., 2024). This revised protocol proved effective in pacu (Criscuolo-Urbinati et al., 2012) and holds potential for application for use in other species (ongoing studies). However, subsequent studies showed that PGF_2α_ levels alone did not fully account for spawning failures in LA migratory species (Kuradomi and Batlouni, 2018; Sato et al., 2023), suggesting the need for further exploration of the mechanisms of actions and effective doses of gonadotropins, steroids, prostaglandins and other substances such as melatonin and cortisol *in vitro*.

The mechanisms underlying FOM and ovulation in native LA fish, such as lambari, remain largely unknown. Classical *in vitro* studies, conducted primarily on species from the Northern Hemisphere (Joy and Singh, 2013; Kagawa et al., 2013) and experimental model species (Lister and Van Der Kraak, 2009; Knight and Van Der Kraak, 2015), have shown that substances involved in FOM and ovulation exhibit varying efficacy mechanism of action across species. This body research has been critical for refining hormonal induction protocols for those species. Since the 1970s, studies have been developed in diverse fish species European (Jalabert et al., 1977; Jalabert and Szöllösi, 1975; Fujimori et al., 2011; Hagiwara et al., 2014; Tang et al., 2016, 2017) have used *in vitro* systems to investigate ovulation failures and the associated mechanisms. Through these investigations, we seek to provide new insights into the hormonal mechanisms regulating ovulation in this species.

## Methods

### Preparation for *In vitro* experimentation and culture conditions

#### Experimental design

##### Experiment 1

The objective of Experiment 1 was to establish baseline conditions for culturing *A. altiparanae* ovarian follicles and to evaluate the induction of FOM and ovulation using protocols adapted from other species. To this end, an initial priming dose was administered *in vivo* to ensure the fish acquired oocyte maturational competence (Patino and Thomas, 1990), and subsequent resolving doses were applied *in vitro*. Previous *in vivo* studies on *A. altiparanae* have shown that while priming doses do not induce germinal vesicle breakdown (GVBD) *in vivo*, they are essential for oocytes to acquire maturational competence (Roza de Abreu et al., 2021, 2022; Ariki et al., 2023).

##### In vivo part of the experiment

Three *A. altiparanae* females were selected based on external characteristics such as rounded bellies and reddish, prominent urogenital papillae (Lira et al., 2018). All females (n=3) were treated with *in vivo* priming injection of carp pituitary extract (CPE) at doses of 0.6 mg/kg body weight.

##### In vitro part of the experiment

Five hours after the priming dose, the females were euthanized, and their ovaries were placed in Hank's balanced salt solution (HBSS) (Vitrocell Embriolife H0345), approximately 25 mL per dish/ovary. The ovaries were rinsed multiple times to remove blood and debris using sterile disposable pipettes. Nine ovarian fragments (approximately 0.1 g each) were collected from each female, yielding a total of 27 fragments, which were individually placed in a sterile, three-compartment Petri dish. Each fragment was placed in an experimental unit (plate compartment) containing 5 mL of medium with the corresponding diluted treatment.

**Figure 1:**
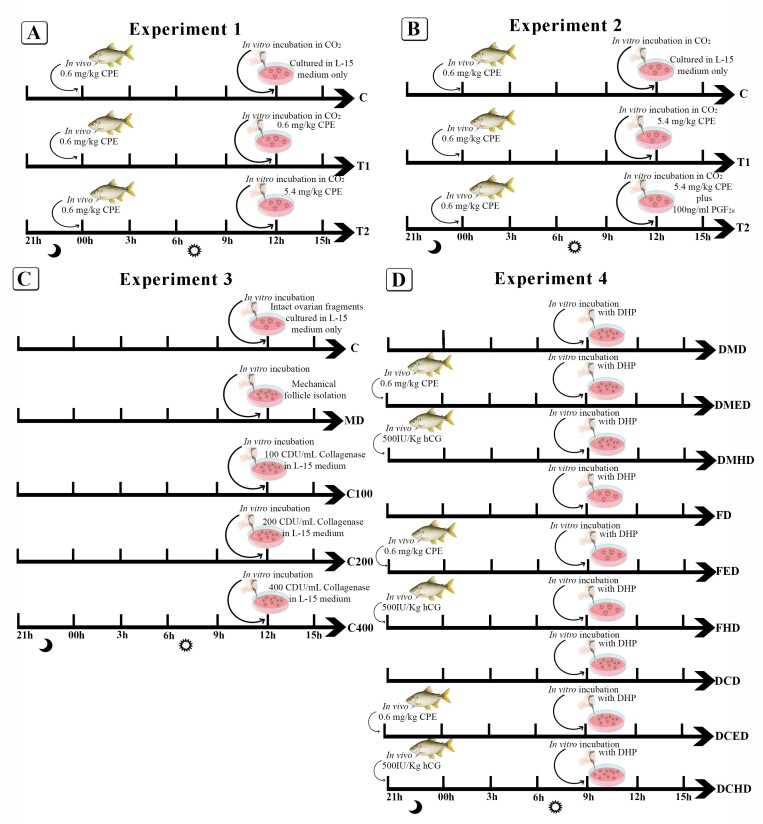
Experimental design of experiments 1 – 4. **A)** Experiment 1: 1st *in vivo* dose with 0.6mg/kg CPE and after 12 hours, *in vitro* incubation in CO2. C: control; T1: treatment 1; T2: treatment 2; **B)** Experiment 2: 1st *in vivo* dose with 0.6mg/kg CPE and after 12 hours, *in vitro* incubation in CO2; C: control; T1: Treatment 1; T2: Treatment 2; **C)** Experiment 3: Test to identify the best way to dissociate the ovarian fragments. C: Control (intact ovarian fragments); MD: Mechanical follicle isolation; C100: 100 CDU/mL Collagenase 100 CDU/mL in L-15 medium; C200: 200 CDU/mL Collagenase 100 CDU/mL in L-15 medium; C400: 400 CDU/mL Collagenase 100 CDU/mL in L-15 medium; **D)** Experiment 4: At the *in vivo* dose, CPE and hCG were tested and all were incubated *in vitro* with DHP. FD: fragment with *in vitro* incubation with DHP; FED: Fragment with 1st *in vivo* dose with EBHC and *in vitro* incubation with DHP; FHD: Fragment with 1st *in vivo* dose with hCG and *in vitro* incubation with DHP; DMD: Mechanical digestion of follicles with *in vitro* incubation with DHP; DMED: Mechanical digestion of follicles with 1st *in vivo* dose with EBHC and *in vitro* incubation with DHP; DMHD: Mechanical digestion of follicles with 1st *in vivo* dose with hCG and *in vitro* incubation with DHP; DCD: Collagenase digestion of follicles with *in vitro* incubation with DHP; DCED: Collagenase digestion of follicles with 1st *in vivo* dose with EBHC and *in vitro* incubation with DHP; DCHD: Collagenase digestion of follicles with 1st dose *in vivo* with hCG and *in vitro* incubation with DHP. Legend: CPE: Carp pituitary extract.

Inside a laminar flow cabinet, the ovarian fragments underwent mild mechanical disintegration by repeatedly aspirating them with a sterile pipette. The HBSS was then replaced with Leibovitz L-15 culture medium (Vitrocell Embriolife L0131) supplemented with gentamicin (0.1 g/L, Sigma BP 918-1). The ovarian fragments were then randomly distributed into three *in vitro* treatments, as described below and in Figure 1A:

- **Control (C):** After receiving an *in vivo* dose of 0.6 mg/kg CPE, the ovarian fragments were cultured in L-15 medium only.
- **Treatment 1 (T1):** Following the *in vivo* priming dose (0.6 mg/kg CPE), fragments received an additional 0.6 mg/kg CPE *in vitro* (the mass of the fragment was considered for the calculation).
- **Treatment 2 (T2):** After the *in vivo* priming dose (0.6 mg/kg CPE), fragments received a higher *in vitro* dose of 5.4 mg/kg CPE.

After overnight incubation (12 hours) in the microprocessor CO_2_ incubator (5% CO2) at a temperature of 26–28ºC, the fragments were separated for the following morning for evaluation.

Follicles of all replicates were removed from the culture medium and subjected to analysis with Serra's Liquid, which allows the presence and position of the nucleus to be visualized. The presence of oocytes without a nucleus, with an eccentric nucleus and with a central nucleus was observed. Part of oocytes were separated for the for histological analysis to compare with stereoscopic evaluation

##### Experiment 2

This experiment followed the same protocol as Experiment 1, but with the addition of cloprostenol (a synthetic analogue of PGF_2α_) *in vitro*, a well-known potent inducer of ovulation in fish (Jalabert and Szöllösi, 1975). Based on *in vivo* studies (Criscuolo-Urbinati et al., 2012), 100 ng/mL of PGF_2α_ was added to Treatment 2. The experimental groups were as follows, as described below and in Figure 1B:

- **Control (C):***In vivo* priming with 0.6 mg/kg CPE and *in vitro* culture with L-15 medium only.
- **Treatment 1 (T1):***In vivo* priming with 0.6 mg/kg CPE and *in vitro* treatment with 5.4 mg/kg CPE.
- **Treatment 2 (T2):***In vivo* priming with 0.6 mg/kg CPE and *in vitro* treatment with 5.4 mg/kg CPE plus 100 ng/ml PGF_2α_.

##### Experiment 3

Due to the lack of clear responses in Experiments 1 and 2, structural modifications were implemented in Experiment 3. CO_2_ was removed from the incubator based on studies suggesting that higher pH levels in the culture medium are beneficial (Welch et al., 2017). Additionally, collagenase (Type II, Sigma-Aldrich) was introduced to aid in follicle isolation (Lacerda et al., 2018), and Trypan Blue staining was used to assess oocyte viability. The experimental design consisted of five groups, each with three replicates, as described below and in Figure 1C:

- **Control (C):** No manipulation (intact 0.1 g ovarian fragments).
- **Treatment 1 (MD):** Mechanical follicle isolation.
- **Treatment 2 (C100):** 100 CDU/mL Collagenase 100 CDU/mL in L-15 medium.
- **Treatment 3 (C200):** 200 CDU/mL Collagenase 100 CDU/mL in L-15 medium.
- **Treatment 4 (C400):** 400 CDU/mL Collagenase 100 CDU/mL in L-15 medium.

After 30 minutes of mechanical or enzymatic treatment, follicles were evaluated for viability (blue for dead, bright for live) and nuclear status.

##### Experiment 4

Considering results obtained in Experiment 3, the experiment 4 tested new ovarian fragment preparation methods, including mechanical and enzymatic incubation without CO_2_. Additionally, human chorionic gonadotropin (hCG) – Sigma-Aldrich, at 500 IU/kg, was introduced as priming dose. For resolving doses, 1 µg/mL 17α,20β-dihydroxy-4-pregnen-3-one (DHP- Sigma-Aldrich) was used for inducing FOM and ovulation induction, replacing pituitaries extracts.

Ovarian fragments from three mature females (3 females and 9 treatments; n=27 fragments) were randomly assigned to different treatments, as outlined in Figure 1D. Twelve hours after the *in vivo* priming doses, ovarian fragments were subjected to *in vitro* culture conditions, including enzymatic digestion with 200 CDU of collagenase/mL for 30 minutes and incubation in L-15 medium, as described in Figure 1D.

All treatments began at 9 p.m., when the *in vivo* priming dose (CPE or hCG) was administered. The following day, 12 hours after the start of the experiment, the *in vitro* phase was initiated. For this, the euthanized females and, after the abdominal incision, 0.2 g ovarian fragments were obtuse and submerged in plastic petri dishes. Each plate initially contains 3 mL of culture medium L-15 (L-15 100%) (L0131, Vitrocell).

Nine experimental treatments were established by combining *in vivo* hormonal protocols and *in vitro* culture strategies:

- **DMD**: (Mechanical dissociation) No *in vivo* hormonal induction. *In vitro* incubation with DHP.
- **DMED**: (Mechanical dissociation) *In vivo* priming dose (0.6 mg/kg CPE). *In vitro* incubation with DHP.
- **DMHD**: (Mechanical dissociation) *In vivo* priming dose (500 IU/kg hCG). *In vitro* incubation with DHP.
- **FD**: (Fragment) No *in vivo* hormonal induction. *In vitro* incubation with DHP.
- **FED**: (Fragment) *In vivo* priming dose (0.6 mg/kg CPE). *In vitro* incubation with DHP.
- **FHD**: (Fragment) *In vivo* priming dose (500 IU/kg hCG). *In vitro* incubation with DHP.
- **DCD**: (Collagenase digestion) No *in vivo* hormonal induction. *In vitro* incubation with DHP.
- **DCED**: (Collagenase digestion) *In vivo* priming dose (0.6 mg/kg CPE). *In vitro* incubation with DHP.
- **DCHD**: (Collagenase digestion) *In vivo* priming dose (500 IU/kg hCG). *In vitro* incubation with DHP.

For mechanical dissociation treatments (DMD, DMED, DMHD), ovarian fragments were manually dissociated in L-15 medium. In whole fragment treatments (FD, FED, FHD), intact ovarian fragment (0.1 g) was maintained in the medium without dissociation. For enzymatic digestion treatments (DCD, DCED, DCHD), ovarian fragments were incubated in L-15 containing 200 UCI/mL type II collagenase on a shaker (27 rpm) for 30 minutes, followed by addition of a stop solution (10% BSA in L-15) to halt enzymatic activity. In all treatments, the DHP (1 µg/mL) was added to the plates. The plates were then placed back on the shaker, maintaining a constant stirring at 27 rpm for 7 hours. After this period, all treatments were evaluated for the success of ovulation induction by counting different types of oocytes: without nucleus, with eccentric nucleus and GVBD. The oocytes without nucleus were considered ovulated, following widely used literature for these analyses (Jalabert and Szöllösi, 1975; Jalabert et al., 1977).

### Ethics

All procedures were conducted at the Fish Reproduction Laboratory (CAUNESP), located in Jaboticabal, SP, Brazil, and were approved by the UNESP Animal Use Ethics Committee under protocol no. 1578/21.

### Animals

The specimens used in this study were adult *A. altiparanae* obtained from captive breeding at the Reproduction Laboratory, CAUNESP, Jaboticabal (21º 15' 17" S, 48º 19' 20" W). The fish were housed in 200m^2^ earthen ponds with a stocking density of 50 fish/m^3^ and a water flow rate of 15-20 L/min. They were fed a commercial diet containing 36% crude protein twice daily until apparent satiety.

## Results

### Experiment 1

**Figure 2:**
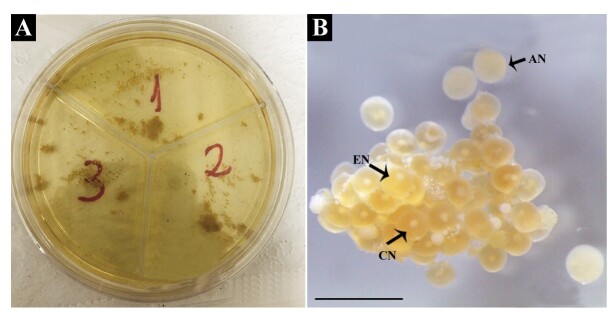
Experiment 1. **A)** Tripartite plate with ovary fragments partially dissociated at the end of *in vitro* step of experiment 1; the yellowish culture medium indicates high acidification; **B)** Cluster of follicles with partial dissociation from treatment T1 (priming dose (0.6 mg/kg CPE) *in vivo* and 0.6 mg/kg carp pituitary extract *in vitro*. All replicas from all treatments showed the same aspect. The Serra liquid technique allowed the identification of the predominance of oocytes with a central nucleus (CN), as well as the presence of eccentric nucleus (EN) and with an absent nucleus (AN). Observe that the overlapping of oocytes does not allow the visualization of many of them. Scale bar: 2mm.

**Figure 3:**
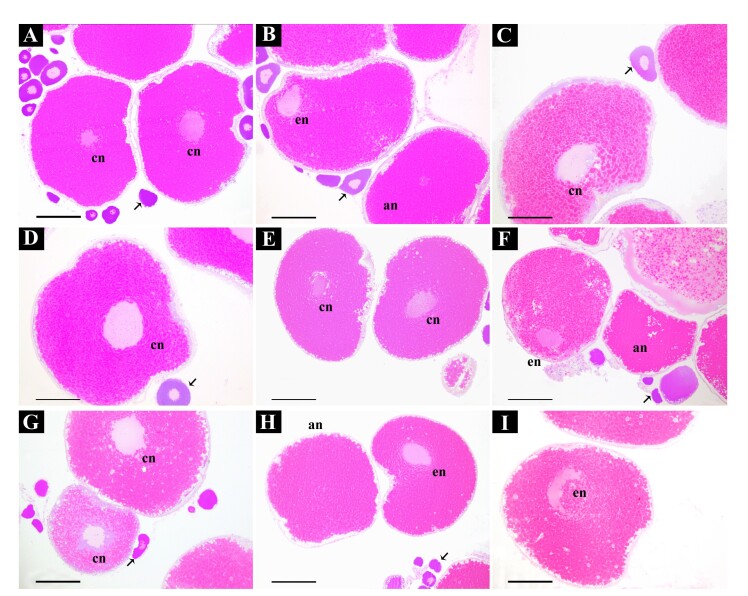
Illustrative images of different treatments of experiment 1. Histological evaluation of follicles at the end of experiment showing the similar distribution of central nucleus (cn), eccentric nucleus (en), and absent nucleus (an) oocytes, among control and treatments. **A-C:** Control; **D-F:** Treatment 1 (0.6 mg/kg CPE *in vitro*); **G-I:** Treatment 2 (*in vitro* dose of 6 mg/kg CPE). Arrow: pre-vitellogenic oocyte. Scale bar: 200μm.

In the general analysis, all culture plates showed intense acidification of the medium, despite being contamination-free (Figure 2A). Mechanical digestion using pipettes did not dissociate the fragments adequately, preventing clear visualization of individual oocytes necessary for nuclear position counting (Figure 2B). However, all fragments contained the three oocyte types: central nucleus (CN), eccentric nucleus (EN), and absent nucleus (AN) (Figure 2B). The images presented are illustrative, as similar patterns were observed across all groups, with minor differences between treatments, but these differences could not be quantitatively analyzed due to the technical challenge of follicle separation. Histological evaluation confirmed the presence of these oocyte types (Figure 3).

### Experiment 2

**Figure 4:**
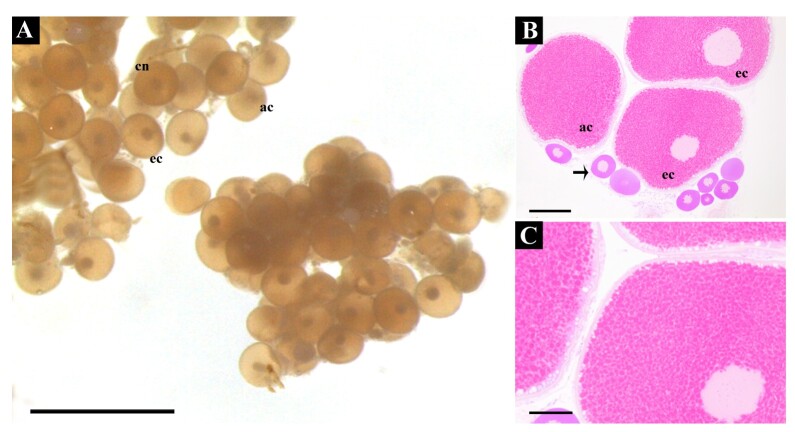
Illustrative images of different treatments of experiment 2. **A)** Cluster of oocytes in Serra's fluid, with inefficient cell separation. Observe that is possible to detect the presence of different type of oocytes; **B)** Through histological analysis, it is also possible to detect and confirm the random and similar presence of different types of oocytes in the different treatments and controls. Cn: central nuclei; ec: excentric nuclei; and an: absent nuclei; **C)** Intact follicular layers and theca in an oocyte with a central nucleus. Scale bar: A: 2mm; B-C: 200mm.

As in Experiment 1, no contamination was observed, but acidification of the culture medium persisted (data not shown). Dissociation of fragments remained inadequate (Figure 4A), and despite introducing prostaglandin in one treatment, no differences in oocyte types were observed across treatments (Figure 4AB). However, histological analysis revealed intact oocytes, including the theca and follicular layers, in all treatments (Figure 4C). Central, eccentric, and absent nuclei oocytes were observed randomLy, with no clear treatment effects. The lack of dissociation of the fragments prevented the counts from being evaluated so that the different types of oocytes could be quantified and compared between treatments, which can be observed in Figure 4A.

### Experiment 3

**Figure 5:**
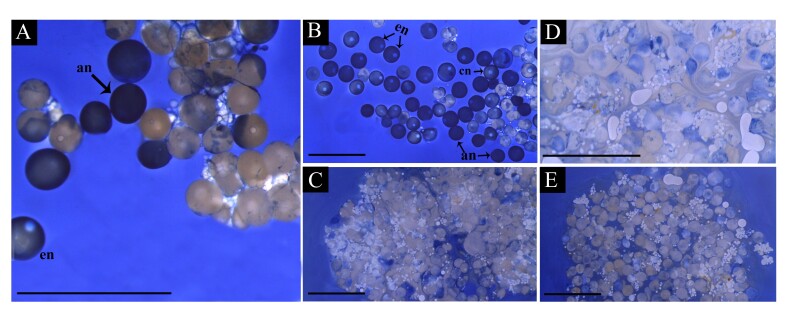
Experiment 3. Evaluation of different types of ovarian follicle isolation. The blue color of some oocytes is due to the method of cell surveillance evaluation based on the use of Trypan blue. **A)** Control, no manipulation. Observe the presence of different oocyte types concerning the blue or bright color and that some oocytes are partially colored. In the same figure is possible to see that germinal vesicle can be central (cn), eccentric (en) or absent (an) and that due to coloration it can be bright (bn) or blue (bn); **B)** Mechanical dissociation of ovarian follicles. Observe that despite the regular aspect of cells, most cells have blue nuclei; **C-E)** Treatments with collagenase. Observe the irregular shape of cells, but despite that, most cells are bright. Treatment with 100 CDU/mL of collagenase type II; D) Treatment with 200 CDU/mL of collagenase type II; E) Treatment with 400 CDU/mL of collagenase type II. Scale bar: A: 2mm; B – E: 5mm.

**Supplementary Figure 1:**
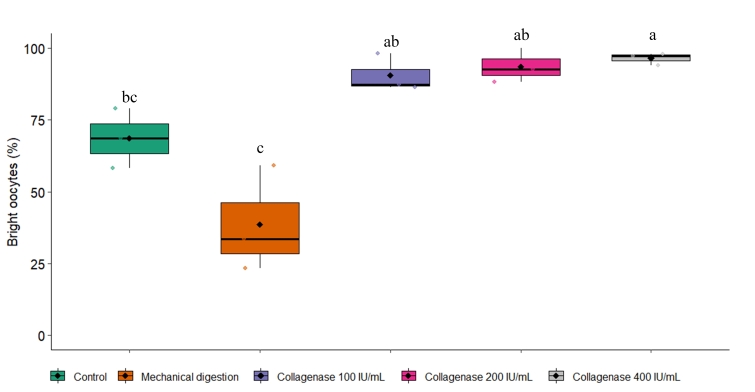
Experiment 3. Survival rate of follicles analyzed using Trypan blue.

Three concentrations of collagenase (100, 200, and 400 IU/mL) were tested (Figure 5). Higher concentrations, especially 400 IU/mL, resulted in a friable appearance of the follicles (Figure 5E), suggesting collagenase digestion disrupted follicular structure. Although most oocytes were not stained with Trypan Blue, indicating viability, the friability of follicles treated with collagenase made these treatments unsuitable (Figure 5A). Follicles disintegrated easily with light handling (Figure 5CE). As discussed later, Trypan Blue may stain only the outer follicle layers without detecting oocyte viability. Cell survival rates are shown in Supplementary Figure 1.

### Experiment 4

**Figure 6:**
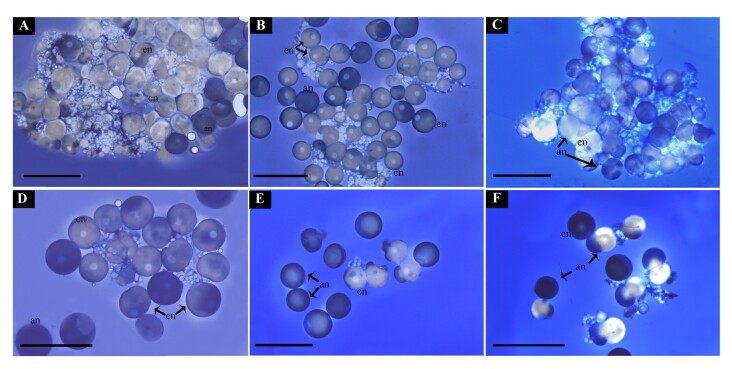
Experiment 4. Obtaining *in vitro* ovulation with 1 μg/mL of 17α,20β-dihydroxy-4-pregnen-3-one (DHP). General appearance of the follicles after the end of experiment 4 of this study. **A)** FD treatment - Entire fragment, without hormonal induction *in vivo*, and incubation *in vitro* with 1 μg/mL DHP ; **B)** FED - Entire fragment with 1st dose *in vivo* with CPE and incubation with 1μg/mL DHP; **C)** FHD - Entire fragment with 1st dose *in vivo* with 500 UI/Kg hCG and incubation with 1μg/mL DHP; **D)** DMD - Follicles from a fish in which mechanical digestion, without hormonal induction *in vivo* and incubation *in vitro* with 1μg/mL DHP; **E)** DMED -Mechanical dissection, with 1st dose *in vivo* with CPE and incubation with 1μg/mL DHP; **F)** DMHD - Mechanical digestion, with 1st dose *in vivo* with 500 UI/Kg hCG and incubation with 1μg/mL DHP. Cn: Central nucleus; ec: excentric nucleus; an: absent nucleus. Scale bar: A, B) 3mm; C, E) 2mm; D) 2,5mm; F) 1mm.

In all groups treated with enzymatic digestion, including controls, post-digestion analysis yielded minimal cellular material, with most samples exhibiting extensive dissolution (data not shown). In the limited instances where follicles were successfully obtained, marked cellular disorganization and loss of cell viability were observed, similar to experiment 3. Whole ovarian fragments were used in some treatments, but this approach proved unsuccessful, as ovulation-inducing substances apparently could not penetrate the follicles to trigger meiosis and ovulation, as will be discussed (Figure 6AC). In contrast, treatments using mechanical digestion achieved adequate follicle separation (Figure 6DF). Notably, the use of DHP, as the resolving dose, specifically in mechanically isolated follicles, effectively induced ovulation, resulting in the release of homogeneous oocytes without germinal vesicles (Figure 6EF).

## Discussion

In this study, we standardized the conditions for maintaining and inducing *A. altiparanae* ovarian follicles to ovulate *in vitro* across four consecutive experiments. To achieve this, we first applied a priming dose *in vivo*, as done in spawning induction protocols for this species (Roza de Abreu et al., 2021, 2022; Ariki et al., 2023). Previous research showed that *A. altiparanae* spawns more effectively when hypophysation doses are fractionated: the priming dose initiates oocyte maturational competence, while the resolving dose induces FOM and ovulation, but only if the priming dose has been administered first (Roza de Abreu et al., 2021, 2022; Ariki et al., 2023). With this in mind, we applied the priming dose *in vivo* following the methodology of Patiño and Thomas (1990) and subsequently administered the resolving dose *in vitro*. This procedure proved effective, as none of the control oocytes (which received only the priming dose *in vivo* and were maintained solely in culture medium *in vitro*) showed signs of GVBD or ovulation. Instead, control oocytes maintained a central nucleus without morphological changes throughout all experiments, confirming the protocol's viability.

Leibovitz’s L-15 medium was selected for follicle culture based on its widespread use in teleosts, including *Danio rerio* (Welch et al., 2017; Mello et al., 2023), *Clarias gariepinus* (Kitanović et al., 2024), *Sebastes schlegelii* (Guo et al., 2024), and *Chaeno gobius annularis* (Kim et al., 2023). Considering the variability in optimal culture conditions among species and methodologies (as reviewed by Hartshorne, 1997), we tested several key factors for *A. altiparanae*, including 5% CO_2_ incubation, ovarian tissue dissociation method (mechanical vs. enzymatic), and the use of intact ovarian fragments versus dissociated follicles. Cell incubators with 5% CO_2_ are commonly used in cell cultures to mimic physiological CO_2_ pressure and concentration levels in tissues, reducing the harmful effects of reactive oxygen species (reviewed in Keeley and Mann, 2019). However, in our initial experiments, CO_2_ incubator led to significant medium acidification. Discontinuing CO_2_ resolved this issue, and in the fourth experiment, successful *in vitro* ovulation was achieved using 1 µg/mL DHP without CO_2_, consistent with findings in zebrafish (Welch et al., 2017). Although it remains unclear whether acidification directly inhibited ovulation, reduced pH is known to impact *in vitro* ovulation processes in fish (Goetz and Nagahama, 1985), warranting further research into the optimal pH environment for *A. altiparanae* follicle incubation. While ovulation was achieved using L-15 medium at standard pH in our study, future studies should also define optimal CO_2_ and O_2_ levels,(reviewed in Samokhin et al., 2022), temperature, osmolarity, and pH conditions for the ovaries of this species to better simulate *in vivo* conditions (reviews in Hartshorne, 1997; Keeley and Mann, 2019).

In the second experiment, we introduced a treatment combining 100 ng/mL PGF_2α_ with the CPE dose to simulate a well-established *in vivo* reproduction protocol (Criscuolo-Urbinati et al., 2012; Sato et al., 2021). However, this approach also did not result in FOM or ovulation. Similar to the first experiment, we observed high acidification of the medium, and the results mirrored those of the control, with no ovulation achieved. These findings suggest that the extract dosage used in the first two experiments may warrant re-evaluation. Although the first and second experiments yielded similar (i.e., non-ovulatory) outcomes, the lack of successful ovulation does not imply that pituitary extracts are ineffective for *in vitro* ovulation in this species; rather, further optimization may be required.

Regarding pituitary extract dosages, when converting mg of CPE per kg of fragment to mg of CPE per mL of medium, the highest dose used in this study was 0.18 µg/mL CPE. In other species, higher doses of pituitary extracts have been effective for inducing *in vitro* hypophyseal activity. For instance, in *Dicentrarchus labrax*, researchers using CPE defined a "pituitary equivalent" (PE) as approximately 3 mg/mL of extract (1 PE/mL) (Sorbera et al., 1999). In their dose-response studies, a single dose ranging from 0.001 to 1 PE was effective in inducing ovulation, with 1 PE achieving nearly 90% oocyte maturation, while 0.001 PE was ineffective in triggering follicle maturation. Given that 1 PE corresponds to 3 mg/mL, this concentration is substantially higher than the 0.18 µg/mL dose used to mimic the resolving dose in lambari in our study. Similarly, in Siberian sturgeon follicles, the minimum effective dose of CPE to achieve 50% maturation was 7.4 µg/mL (Goncharov et al., 2001), which is ten times higher than the maximum dose tested in our study. These comparisons highlight the significant variability in the potency of pituitary extracts across species, suggesting that dose optimization is species-specific for effective *in vitro* maturation.

When comparing different substances, both dosage and latency period must be considered. In this study, we applied the same experimental duration for treatments with both pituitary extracts and DHP, despite their distinct latency periods *in vitro*. Typically, gonadotropins require longer to induce ovulation than DHP. For example, in *Discentraxus labrax*, the latency period for exogenous gonadotropins and DHP was approximately 45 and 15 hours, respectively (Sorbera et al., 1999). In Siberian sturgeon, a 50% ovulation rate with pituitary extracts was observed after about 36-38 hours of incubation (Goncharov et al., 2001). In contrast, the present study evaluated the response to CPE only 7 hours post-dose, aligning with the species’ *in vivo* ovulation timeline (Roza de Abreu et al., 2025). Therefore, future studies should extend the incubation period to fully assess the ovulation-inducing effects of pituitary extracts in lambari, adjusting both dose and incubation time.

This perspective is also relevant to the absence of ovulation observed with 100 ng/mL PGF_2α_ in the third experiment. The concentration of PGF_2α_, derived from *in vivo* studies considering total body mass (Criscuolo-Urbinati et al., 2012), is relatively low for *in vitro* cultures. In fact, Jalabert and Szöllösi (1975) demonstrated that *in vitro* ovulation in trout could be achieved with PGF_2α_ concentrations ranging from 1,000 to 5,000 ng/mL, which are 10 to 50 times higher than the dose used in our study. These findings strongly suggest that the dose of PGF_2α_ employed in our experiment may have been insufficient to trigger ovulation, underscoring the need for dose optimization in *in vitro* protocols.

In the present study, Trypan Blue staining proved more effective for visualizing the outer follicular layers than for reliably assessing follicle viability, as staining was inconsistent and not clearly associated with oocyte death. Similar limitations were reported by Santos et al. (2017) in evaluate tambaqui follicle viability. By the fourth experiment, mechanical digestion was the most effective method for isolating lambari follicles. Notably, in this experiment, we observed promising signs of *in vitro* ovulation exclusively in treatments combining mechanical digestion with DHP. These results, helped confirm that the blue coloration of oocytes is not indicative of dead follicles. Although collagenase digestion produced morphologically brighter oocytes, it failed to yield viable follicles.

Given that DHP and other maturation-inducing steroids (MIS) typically induce FOM and ovulation more rapidly and effectively *in vitro* than gonadotropins or pituitary extracts (Jalabert and Szöllösi, 1975; Patiño and Thomas, 1990; Sorbera et al., 1999; Goncharov et al., 2001; Welch et al., 2017), we opted to replace CPE with 1 µg/mL DHP as the resolving dose in the fourth experiment. At this stage, improved experimental conditions were also established, opting to use DHP as an *in vitro* steroid inducer, similar to the application of prostaglandins in *Anguilla japonica* (Kagawa et al., 2003) or DHP in *Perca fluviatilis* (El Mohajer et al., 2021). Our study corroborates previous findings by Jesus et al. (2017), confirming DHP as a MIS for lambari, though the involvement of other MIS cannot be ruled out. DHP is widely recognized as a key MIS for many fish species (Takahashi et al., 2019), but additional investigations are needed to fully elucidate the specific MIS involved in lambari oocyte maturation. Based on our findings, mechanical digestion of ovarian fragments combined with an *in vivo* priming dose and in *vitro* DHP application as a resolving dose effectively induces *in vitro* ovulation. These findings provide a foundation for optimizing *in vitro* ovulation protocols in lambari and offer a potential framework for similar advancements in other fish species.

## Conclusion

This study established a viable protocol to induce ovulation *in vitro* in *A. altiparanae*, highlighting the role of DHP and the efficiency of mechanical digestion. Future studies should focus on optimizing hormone doses and incubation parameters to further enhance the method's efficacy and applicability.

## Data Availability

Research data is only available upon request.

## Supplementary Material

Supplementary material accompanies this paper.
